# Reactive Self-Collision Avoidance for a Differentially Driven Mobile Manipulator

**DOI:** 10.3390/s21030890

**Published:** 2021-01-28

**Authors:** Keunwoo Jang, Sanghyun Kim, Jaeheung Park

**Affiliations:** 1 Graduate School of Convergence Science and Technology, Seoul National University, Seoul 08826, Korea; jkw0701@snu.ac.kr; 2 Korea Institute of Machinery and Materials (KIMM), Daejeon 34103, Korea; kim87@kimm.re.kr; 3 Advanced Institute of Convergence Technology (AICT), Suwon 16229, Korea

**Keywords:** self-collision avoidance, whole-body motion planning and control, mobile manipulation

## Abstract

This paper introduces a reactive self-collision avoidance algorithm for differentially driven mobile manipulators. The proposed method mainly focuses on self-collision between a manipulator and the mobile robot. We introduce the concept of a distance buffer border (DBB), which is a 3D curved surface enclosing a buffer region of the mobile robot. The region has the thickness equal to buffer distance. When the distance between the manipulator and mobile robot is less than the buffer distance, which means the manipulator lies inside the buffer region of the mobile robot, the proposed strategy is to move the mobile robot away from the manipulator in order for the manipulator to be placed outside the border of the region, the DBB. The strategy is achieved by exerting force on the mobile robot. Therefore, the manipulator can avoid self-collision with the mobile robot without modifying the predefined motion of the manipulator in a world Cartesian coordinate frame. In particular, the direction of the force is determined by considering the non-holonomic constraint of the differentially driven mobile robot. Additionally, the reachability of the manipulator is considered to arrive at a configuration in which the manipulator can be more maneuverable. In this respect, the proposed algorithm has a distinct advantage over existing avoidance methods that do not consider the non-holonomic constraint of the mobile robot and push links away from each other without considering the workspace. To realize the desired force and resulting torque, an avoidance task is constructed by converting them into the accelerations of the mobile robot. The avoidance task is smoothly inserted with a top priority into the controller based on hierarchical quadratic programming. The proposed algorithm was implemented on a differentially driven mobile robot with a 7-DOFs robotic arm and its performance was demonstrated in various experimental scenarios.

## 1. Introduction

A mobile manipulator, which is a manipulator mounted on a mobile robot, has infinite workspace based on the mobility offered by the mobile robot. Furthermore, the degrees of freedom (DOFs) of the mobile robot typically provide the mobile manipulator with redundancy with respect to the tasks such as end-effector trajectory tracking. By utilizing these properties, a mobile manipulator can perform complex and diverse tasks such as painting [1], production [2], and manufacturing [3]. However, to perform these complex tasks in dynamic and unstructured environments, one of the most critical capabilities for a mobile manipulator is to detect self-collision by using proprioceptive or exteroceptive sensors and reactively generate sensor-based avoidance motion. In this paper, we present a new algorithm for reactive self-collision avoidance for a differentially driven mobile manipulator.

### 1.1. Related Works

Self-collision can be avoided using either offline or online motion generation. Planning collision-free motion is typically implemented offline, whereas online motion generation is mainly embedded with the controllers. First, in the field of motion planning, Kuffner et al. [4] proposed a motion planning algorithm to compute dynamically stable and collision-free trajectories for humanoid robots based on Rapidly-exploring Random Trees (RRT). Oriolo and Mongillo [5] proposed a randomized planner that resolves the redundancy of non-holonomic mobile manipulator. The planner allows a mobile robot to be located within a compatible region for a given end-effector position so that the inverse kinematics solutions for the manipulator can be derived. Regarding pose constraints on the end-effector, Berenson et al. [6] developed Constrained Bi-directional RRT (CBiRRT) that plans the trajectory by projecting sampled position onto Task Space Region (TSR). Burget et al. [7] proposed a planning framework called Bi-directional Informed RRT${}^{*}$(BI${}^{2}$RRT${}^{*}$) that can efficiently obtain optimal paths for mobile manipulation under task space constraints. Furthermore, Welschehold et al. [8] proposed a motion planner for the mobile manipulator, which exploited the concept of dynamical system approach for obstacle avoidance and the concept of inverse reachability for approximating the inner and outer boundaries of workspace. Kang et al. [9] proposed a sampling-based method that efficiently explores whole-body configuration space by sampling more around a region close to obstacles. However, these methods are difficult to implement in unstructured and dynamic environments because trajectories may have to be regenerated in real time. Additionally, their computational cost is relatively high for robots with large number of DOFs, such as humanoids or mobile manipulators.

To overcome these limitations, many reactive methods have been proposed to detect and avoid self-collision in real time. Our proposed method belongs to this category. Seto et al. [10,11] designed the outer parts of links as elastic elements so that the reaction forces are generated between elastic elements when links move close to each other. In [12,13], motions for self-collision avoidance were generated based on the gradient of a cost function related to the distances between links. Dariush et al. [14] penalized joint motions using the inverse matrix of weighted Jacobian based on the gradient of a collision function. Fang et al. [15] proposed a method for generating relative motion between the links using an inequality task. Quiroz-Omaña et al. [16] designed a distance function and converted it to the form of inequality constraint to generate relative acceleration. However, these methods are not applicable to non-holonomic mobile manipulators because they were developed for holonomic systems. Specifically, the methods may not instantaneously generate motion of the mobile robot in a certain direction because the non-holonomic constraint is not considered [17,18]. On the other hand, Dietrich et al. [19,20] proposed a repulsive force-based approach with an efficient damping design and extended continuous null space projection method. Sugiura et al. [21] proposed a method using only 1-DOF repulsive force while dynamically swapping the priority of the tasks. Gonon et al. [22] proposed a modified artificial potential field that includes a viscous damping term to dissipate energy and enforces a limit on the repulsive forces, thus can prevent the repulsive force from oscillating with high frequency. Although repulsive force-based methods are conservative and effective solution for avoiding collisions, the methods repel two proximate links and thus the modified motion may be farther away from the reference motion more than necessary. Lei et al. [23] proposed a method for humanoid dual-arm robot that generates smooth repulsive velocity based on designing the links as discretized spheres. However, the method does not consider self-collision between the manipulator and mobile robot.

### 1.2. Overview of This Paper

**Figure 1:**
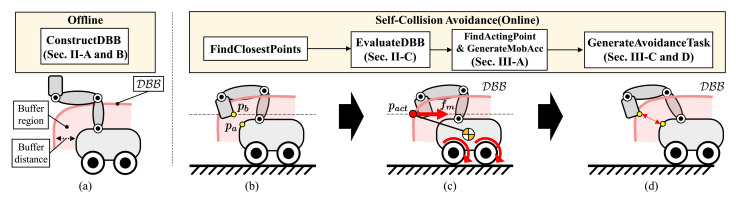
Overview of the proposed algorithm. (**a**) For each link colliding with the mobile robot, the DBB is defined as the boundary of the buffer region enclosing the mobile robot. (**b**) For example, when the distance between the manipulator and mobile robot is less than the buffer distance, the two closest points ($p_{a}$, $p_{b}\in R^{3}$) are calculated. (**c**) To avoid self-collision, our strategy is to exert a force $f_{m}\in R^{3}$ on the mobile robot to ensure that the manipulator lies outside the DBB. To accomplish this goal, a point on the DBB is selected as the acting point $p_{\mathrm{act}}\in R^{3}$ for the force after evaluating the 3D points on the DBB as scores. The target direction of the force is toward the closest point $p_{b}$ on the manipulator. (**d**) To realize the desired force and resulting torque, we construct the avoidance task by converting them to the accelerations for the mobile robot. As the task is continuously inserted into the controller, self-collision between the manipulator and mobile robot can be avoided.

In this paper, we propose a new self-collision avoidance algorithm for a differentially driven mobile manipulator. Our focus is on avoiding self-collision between a manipulator and mobile robot. Our goal is to generate a motion in which the manipulator can avoid self-collision without modifying its reference motion. To this end, we propose the concept of the DBB, a border of the buffer region surrounding the mobile robot. This region has a thickness equal to the buffer distance (see Figure 1a). When the manipulator and the mobile robot are close to each other, meaning their distance is less than the buffer distance (see Figure 1b), our strategy is to position the manipulator outside the DBB by moving the mobile robot. This can be accomplished by generating a force exerted on the mobile robot because the DBB is attached to the mobile robot and moves with it (see Figure 1c). Therefore, the manipulator can avoid self-collision with the mobile robot without modifying reference motion of the manipulator (see Figure 1d).

Especially, the direction of force is determined by considering the following two factors. First, the singularity of the differentially driven mobile robot due to non-holonomic constraint is considered in order not to lose the controllability of the mobile robot. Second, we consider the reachability of the manipulator, which is a representation of the robot’s workspace with information regarding pose quality. Because the direction of force is determined to enhance the reachability, the resultant configuration of the robot can secure a large workspace of the manipulator.

To implement the proposed algorithm on a robot, an avoidance task is constructed by combining two types of motions depending on whether a link collides with the mobile robot or not. First, for a link pair including the mobile robot, the desired force and resulting torque are generated and converted into accelerations of the mobile robot. Second, for a link pair that does not includes the mobile robot, 1-DOF acceleration is generated in the direction in which that the distance between the closest points of the link pair increases. The avoidance task is then constructed by stacking two types of accelerations and their Jacobian matrices. The task is inserted continuously with the highest priority depending on the distances between the link pairs by using a controller based on Hierarchical Quadratic Programming (HQP) with the continuous task transition algorithm [24,25]. The HQP can handle multiple tasks with strict priorities, Stack of Tasks (SoT).

The remainder of this paper is organized as follows. First, Section 2 details the DBB and the computation of its score for deriving the desired force. Second, Section 3 explains our overall strategy for self-collision avoidance. Next, Section 4 describes the experimental validation of the proposed strategy. Finally, the paper is concluded in Section 5. To enhance readability of this paper, Table 1 lists the symbols used in this paper and their corresponding definitions. Bold Roman letters denote vectors and matrices while normal Roman letters denote real numbers.

## 2. Distance Buffer Border and Its Score Computation

This section introduces the concept of the DBB for generating a force that avoids self-collision between a manipulator and mobile robot. Additionally, we describe how to compute the score of the DBB to determine the direction and magnitude of the force. In Section 2.1, all link pairs that can potentially collide with each other are identified based on the collision model and kinematic information of the robot. Next, for the link pairs including the mobile robot, we define the DBB in Section 2.2. Finally, two factors are introduced to calculate the scores of the points on the DBB in Section 2.3.

### 2.1. Identification of Potentially Colliding Link Pairs

**Figure 2:**
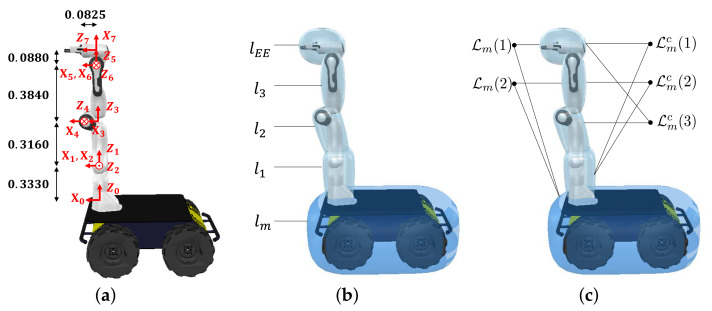
Our mobile manipulator system consists of a four-wheel differentially driven mobile robot called Husky (Clearpath Robotics. Co.) and 7-DOFs manipulator called Panda (Franka Emika. Co.). (**a**) Kinematic structure of the manipulator is shown with the scale of meter; (**b**) the simplified collision models of the robot consist of five links; (**c**) based on the collision models and joint range of the manipulator, all link pairs that potentially collide with each other are identified. $L_{m}\left(i\right)$ denotes the link pair including the mobile robot, whereas $L_{m}^{c}\left(j\right)$ denotes the link pair not including the mobile robot.

To decrease the computational cost for checking self-collision, simplified collision models are designed by using the convex shapes based on the kinematic structure of the manipulator. Figure 2a,b shows the kinematic structure of our robot and collision models of the robot, respectively. Utilizing these models and the joint position ranges of the manipulator, the link pairs which never collide with each other can be precomputed by randomly sampling the joint position of the manipulator. From this analysis, the link pairs potentially colliding with each other are identified. The set of the link pairs is defined as follows (note that $l_{2}$ never collides with $l_{m}$ because the range of the 2nd joint position of the manipulator is physically limited from −101 deg to 101 deg).

(1) $$\begin{array}{c} L=\{<l_{m},l_{EE}>,<l_{m},l_{3}>,<l_{1},l_{EE}>,<l_{1},l_{3}>,<l_{2},l_{EE}>\}, \end{array}$$

where $l_{•}$ denotes an individual link in Figure 2b. Because the avoidance motion is generated differently depending on whether or not the link pair includes the mobile robot $l_{m}$, the set L is divided into two subsets, namely $L_{m}$ and its complement $L_{m}^{c}$, and shown in Figure 2c as follows.

(2) $$\begin{array}{cc} L_{m} & =\{<l_{m},l_{EE}>,<l_{m},l_{3}>\}\\ L_{m}^{c} & =\{<l_{1},l_{EE}>,<l_{1},l_{3}>,<l_{2},l_{EE}>\}. \end{array}$$

In (2), we denote each element of subset as $L_{m}\left(i\right)$ and $L_{m}^{c}\left(j\right)$ respectively. Therefore, to avoid self-collision, we generate a force on the mobile robot for $L_{m}$ as discussed in Section 3.1 and 1-DOF repulsive acceleration for $L_{m}^{c}$ as discussed in Section 3.2.

### 2.2. Distance Buffer Border

Our avoidance strategy is to move the mobile robot in order to place the manipulator outside a region surrounding the mobile robot with a thickness equal to the buffer distance. To this end, we define a border of the region as the DBB of $L_{m}$. Geometrically, the DBB represents a group of 3D points located away from the mobile robot $l_{m}$ by the buffer distance.

**Figure 3:**
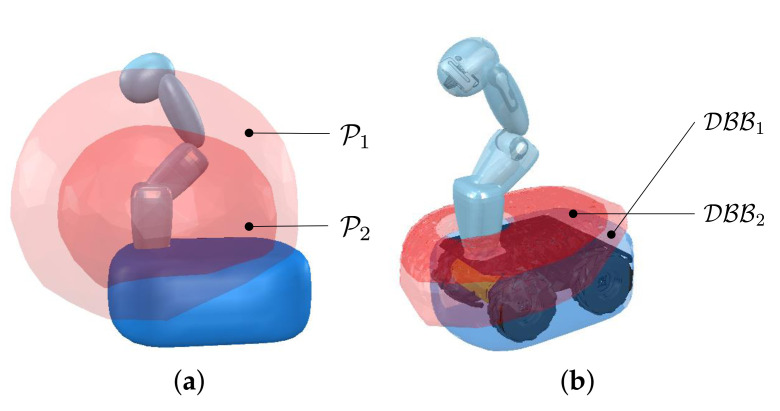
Visualization of $P_{i}$ and ${\mathrm{DBB}}_{i}$ for $L_{m}\left(i\right)$. (**a**) Red volume represents $P_{i}$ which is a point set around the mobile robot; (**b**) red hyperplanes represent the distance buffer borders of $L_{m}$. The buffer distance $d_{b}$ is set to 0.15 m and the tolerance ϵ is set to 0.01 m.

Algorithm 1 describes the construction of the DBB in detail and is implemented offline. The input for the algorithm is the set of stored points on the manipulator’s link for $L_{m}$. For each link pair in $L_{m}$, the two closest points are calculated after randomly sampling the joint positions of the manipulator. The point on the link of the manipulator is then stored to the set. This process repeats until the set contains a sufficient number of points. For the *i*-th link pair in $L_{m}$, each set is denoted by $P_{i}$ as shown in Figure 3. The algorithm operates as follows.
**Algorithm 1** ConstructDBB**Input:** $P_{i}$ : a set of points on the manipulator’s link for $L_{m}\left(i\right)$**Output:** ${\mathrm{DBB}}_{i}$ : distance buffer border of $L_{m}\left(i\right)$1:**for** each $p_{i}$ in $P_{i}$ **do**2:    $d_{m}\leftarrow \mathrm{DistanceToMobile}\left(p_{i},l_{m}\right)$3:    **if** $∥d_{m}-d_{b}∥\leq \epsilon$ **then**4:        Store $p_{i}$ in ${\mathrm{DBB}}_{i}$5:    **end if**6:**end for**

First, for each point $p_{i}$ of $P_{i}$, the DistanceToMobile function calculates the distance between $p_{i}$ and the mobile robot (see Line 2). Second, if the distance is within a bounded range, then the point $p_{i}$ is stored in the DBB (see Line 3–4). After these procedures are repeated, the DBB is constructed as shown in Figure 3b and defined as follows:

(3) $$\begin{array}{cccc} \;\;\; & \;\;\;\;\;\; & \;\;\; & {\mathrm{DBB}}_{i}∋{}^{\forall }p_{i}\\ \; & \;s.\;t.\; & \; & ∥d_{m}-d_{b}∥\leq \epsilon ,p_{i}\in P_{i} \end{array}$$

where ${\mathrm{DBB}}_{i}$ denotes the DBB for $L_{m}\left(i\right)$, $p_{i}\in R^{3}$ is the position on the link of the manipulator, $d_{m}$ is the minimum distance between $p_{i}$ and $l_{m}$, $d_{b}$ is buffer distance, and ϵ is tolerance value. Even though Figure 3 shows the DBB for our robot, the DBB for other differentially driven mobile manipulator can be obtained if kinematic structure and collision models of the robot are given. To accomplish our strategy, we generate a force on the mobile robot based on the DBB. The direction of the force is defined to begin at a point on the DBB and head toward a point on the manipulator. The point on the DBB becomes the acting point of the force as shown in Figure 1c. In the following subsection, we propose a score for evaluating every point on the DBB to select the acting point.

### 2.3. Evaluation of Distance Buffer Border

To select the point on the DBB that satisfies the desired capabilities of the force, the DBB is evaluated based on a score consisting of two factors: the singularity of the differentially driven mobile robot and the reachability of the manipulator.

#### 2.3.1. Singularity of the Differentially Driven Mobile Robot

**Figure 4:**
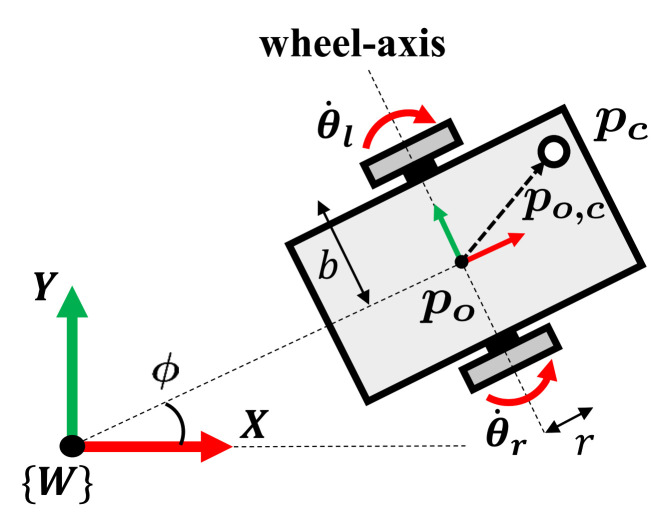
Schematic drawing of the differentially driven mobile robot. $p_{c}$ is the control point of the mobile robot in the world frame $\left\{W\right\}$ and $p_{o,c}$ is the planar vector from the center of the mobile robot, $p_{o}$, to $p_{c}$ in $\left\{W\right\}$. *b* and *r* are the distance between the wheel and center of the mobile robot and the radius of the wheel, respectively. ${\dot{\theta }}_{r}$ and ${\dot{\theta }}_{l}$ are the spinning velocities of the right and left wheel, respectively.

First, the singularity of the differentially driven mobile robot is considered to prevent the force from generating the motion of the non-holonomic mobile robot along the singular direction. Figure 4 shows the kinematic modeling of two-wheel differentially driven mobile robot which simplifies that of four-wheel differentially driven mobile robot [26]. The differentially driven mobile robot is subject to a constraint in terms of the velocity as follows.

(4) $$-{\dot{x}}_{o}sin\left(\phi \right)+{\dot{y}}_{o}cos\left(\phi \right)=0$$

where ${\dot{x}}_{o}$ and ${\dot{y}}_{o}$ are planar velocity of the center of the mobile robot and ϕ is the heading angle of the robot from the X-axis in the world frame as shown in Figure 4. Physically, (4) means that there is no velocity component parallel to the wheel-axis at the center of the differentially driven mobile robot. The constraint is non-integrable, thus termed as non-holonomic constraint [27,28].

The velocity relationship between the control point and the configuration of the differentially driven mobile robot is given by

(5) $${\dot{p}}_{c}=J_{c}\left(q_{c}\right){\dot{q}}_{b},$$

where ${\dot{p}}_{c}={\left[\begin{array}{cc} {\dot{x}}_{c} & {\dot{y}}_{c} \end{array}\right]}^{T}\in R^{2}$ is the planar velocity of the control point of the mobile robot. ${\dot{q}}_{b}={\left[\begin{array}{cc} {\dot{\theta }}_{r} & {\dot{\theta }}_{l} \end{array}\right]}^{T}\in R^{2}$ is spinning velocity of the wheels and the subscripts *r* and *l* of $\dot{\theta }$ denote the right and left wheel, respectively. $J_{c}\left(q_{c}\right)\in R^{2\times 2}$ is Jacobian matrix given by

(6) $$\begin{array}{c} J_{c}\left(q_{c}\right)=\left[\begin{array}{cc} c(b\cos\phi -y_{o,c}) & c(b\cos\phi +y_{o,c})\\ c(b\sin\phi +x_{o,c}) & c(b\sin\phi -x_{o,c}) \end{array}\right], \end{array}$$

where c=r/2b, *r* is the radius of the wheel, *b* is the distance between the wheel and the center of the mobile robot, and $q_{c}={\left[\begin{array}{cc} p_{o,c} & \phi \end{array}\right]}^{T}$. $p_{o,c}=\left[\begin{array}{cc} x_{o,c} & y_{o,c} \end{array}\right]$ are the coordinates of the control point from the center of the mobile robot in the global frame and ϕ is the orientation of the mobile robot.

To identify the singularity, we derive the determinant of the product of the Jacobian matrix $J_{c}\left(q_{c}\right)$ as

(7) $$det\left(J_{c}{J_{c}}^{T}\right)=4b^{2}c^{4}{\left(x_{o,c}\cos\phi +y_{o,c}\sin\phi \right)}^{2}.$$

From (7), the Jacobian matrix $J_{c}\left(q_{c}\right)$ loses rank when

(8) $$x_{o,c}\cos\phi +y_{o,c}\sin\phi =0.$$

Geometrically, the left side of (8) represents the distance between the control point and the line of the wheel-axis. As the value of (8) tends to zero, meaning the control point is located on the wheel-axis, the control point of the differential-driven mobile robot cannot instantaneously move along the wheel-axis [29].

**Figure 5:**
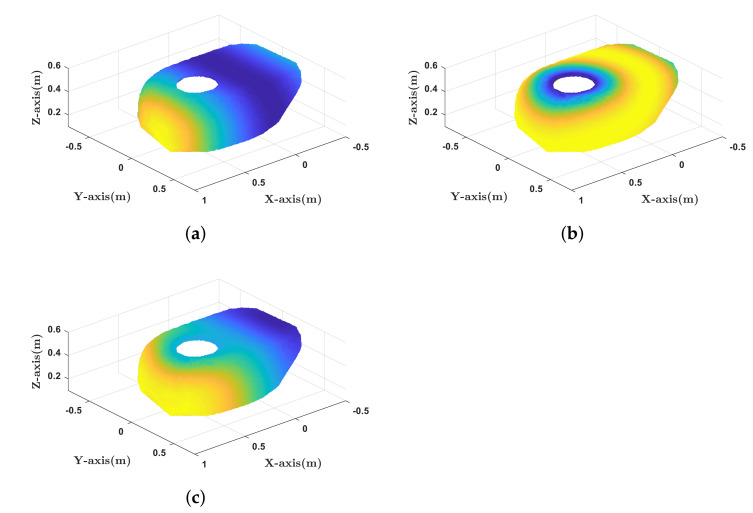
All the points in ${\mathrm{DBB}}_{1}$ are colored depending on (**a**) the determinant value in (7), (**b**) the reachability in (9), and (**c**) the score in (10) (yellow: high, blue: low).

Thus, assuming that each point $p_{i}$ of ${\mathrm{DBB}}_{i}$ is set to the control point of the mobile robot, we can measure how close it is to the singularity by setting $q_{c}={\left[\begin{array}{ccc} x_{i} & y_{i} & 0 \end{array}\right]}^{T}$ where $x_{i}$ and $y_{i}$ are the coordinates of $p_{i}$ along X-axis and Y-axis, respectively. For our robot, *r* is set to 0.165 m and *b* is set to 0.51 m. In Figure 5a, the value of (7) for each point on ${\mathrm{DBB}}_{1}$ is computed. As shown in Figure 5a, the determinant value is symmetric about X-axis.

#### 2.3.2. Reachability of the Manipulator

**Figure 6:**
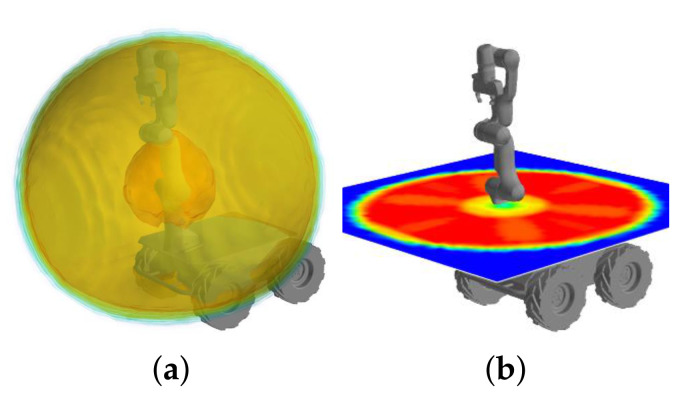
Visualization of reachability shown in OPENRAVE [31]. (**a**) The contour of reachability of the end-effector; (**b**) the reachability cut by a horizontal plane at the base of the manipulator is colored (right, red: high, blue: low).

Second, the reachability of the manipulator is considered in order for the force to place the manipulator in the suitable workspace. The reachability is defined as the density of Inverse Kinematics (IK) solutions for the pose of the end-effector [30]. Reachability is computed by uniformly sampling the pose of the end-effector over the entire workspace and recording the number of IK solutions for each pose. The reachability of our robot is illustrated in Figure 6. One can see that the value of reachability increases and then decreases as the pose of the end-effector moves outwards from the base of the manipulator. Based on this observation, reachability can be expressed as a scalar concave function of the distance from the base of the manipulator. Among the various types of concave functions, the following second-order polynomial function was selected in this paper.

(9) $$R\left(p_{i}\right)=-A(∥p_{i}-p_{\mathrm{base}}{{∥}_{2}-B)}^{2}+C,$$

where $R\left(p_{i}\right):R^{3}\to R^{+}$ maps points on the DBB to reachability values, $p_{\mathrm{base}}\in R^{3}$ is the position of the base of the manipulator, and A,B,C are positive coefficients of the polynomial. Based on the reachability data in Figure 6, we set *A* to 525.9 /m${}^{2}$, *B* to 0.575 m, and *C* to 100. Figure 5b presents the reachability value for each point on ${\mathrm{DBB}}_{1}$. Although reachability is originally defined for the pose of the end-effector, the reachability of other link of the manipulator can also be obtained using kinematics information.

#### 2.3.3. Score of the DBB

We compute a score for every point on the DBB denoted as $S(p_{i})\in R$. A score is expressed as

(10) $$S\left(p_{i}\right)=sign\left(x_{i}\right)det\left(J_{c}{J_{c}}^{T}\right)R\left(p_{i}\right)$$

where

(11) $$sign\left(x\right)=\left\{\begin{array}{cc} 1 & x\geq 0\\ -1 & x<0. \end{array}\right.$$

Note that the function sign ensures that the DBB has a point of global maximum score as shown in Figure 5c.

## 3. Self-Collision Avoidance Algorithm

In this section, we explain how to avoid self-collision for the differentially driven mobile manipulator. Algorithm 2 details the procedure. First, the FindClosestPoints function calculates the closest pair of points for each link pair. The link pair in $L_{m}$ then generates the acceleration of the mobile robot, whereas the acceleration of the manipulator is generated for $L_{m}^{c}$. For the subset $L_{m}$, the FindActingPoint function determines the acting point of the force based on the computation for the score of the DBB. Next, the GenerateMobAcc function generates the force and the resulting torque exerted on the mobile robot and converts them to the linear and angular acceleration of the mobile robot as ${\ddot{\underline{x}}}_{m}\in R^{2}$ (see Line 5–6 and Section 3.1). On the other hand, for the subset $L_{m}^{c}$, the GenerateRepAcc function generates a 1-DOF repulsive acceleration which pushes the two proximal links of the manipulator away from each other and stacks the accelerations for *k* link pairs of $L_{m}^{c}$ as ${\ddot{\underline{x}}}_{r}\in R^{k}$ (see Line 10 and Section 3.2). Then, the GenerateAvoidanceTask function combines these accelerations and constructs the task for avoiding self-collision, $T_{sca}$, as an equality task (see Line 13 and Section 3.3). Finally, the UpdateSoT function inserts the task $T_{sca}$ as a top priority in the original SoT by using the continuous task transition scheme, as summarized in Line 14–15 and Section 3.4. In the following subsections, each function in the Algorithm 2 is described in detail.
**Algorithm 2** Self-Collision Avoidance**Input:** A set of link pairs $L=L_{m}\cup L_{m}^{c}$; DBBs of the links DBB1:**while** IsControl() **do**2:    UpdateKinematics(q)// Avoidance between mobile robot and manipulator3:    **for** each $L_{m}\left(i\right)$ **do**4:        $\left(p_{a,i},p_{b,i}\right)\leftarrow \mathrm{FindClosestPoints}\left(L_{m}\left(i\right)\right)$// $p_{a,i}$ on mobile robot, $p_{b,i}$ on manipulator5:        $p_{\mathrm{act},i}\leftarrow \mathrm{FindActingPoint}\left(q,p_{b,i},{\mathrm{DBB}}_{i}\right)$6:        ${\ddot{\underline{x}}}_{m}\leftarrow \mathrm{GenerateMobAcc}\left(p_{\mathrm{act},i},p_{b,i}\right)$7:    **end for**// Avoidance between links of manipulator8:    **for** each $L_{m}^{c}\left(j\right)$ **do**9:        $\left(p_{a,j},p_{b,j}\right)\leftarrow \mathrm{FindClosestPoints}\left(L_{m}^{c}\left(j\right)\right)$10:        ${\ddot{\underline{x}}}_{r}\leftarrow \mathrm{GenerateRepAcc}\left(p_{a,j},p_{b,j}\right)$11:    **end for**// Insert the task continuously to the controller12:    **if** $∥{\ddot{\underline{x}}}_{m}{∥}_{2}>0$**or**$∥{\ddot{\underline{x}}}_{r}{∥}_{2}>0$ **then**13:        $T_{sca}\leftarrow \mathrm{GenerateAvoidanceTask}\left({\ddot{\underline{x}}}_{m},{\ddot{\underline{x}}}_{r}\right)$14:        $SoT\leftarrow \mathrm{UpdateSoT}(T_{sca})$15:        u←HQPSolver(SoT) // See (29)16:    **else**17:        u←HQPSolver(SoT) // See (26)18:    **end if**19:**end while**

### 3.1. Generation of the Acceleration for the Mobile Robot

This subsection describes the generation of the force. The direction of the force is designed to start from the selected point on the DBB and head to the closest point on the manipulator. Thus, we focus on how to select the acting point of the force that satisfies the following requirements.

**Figure 7:**
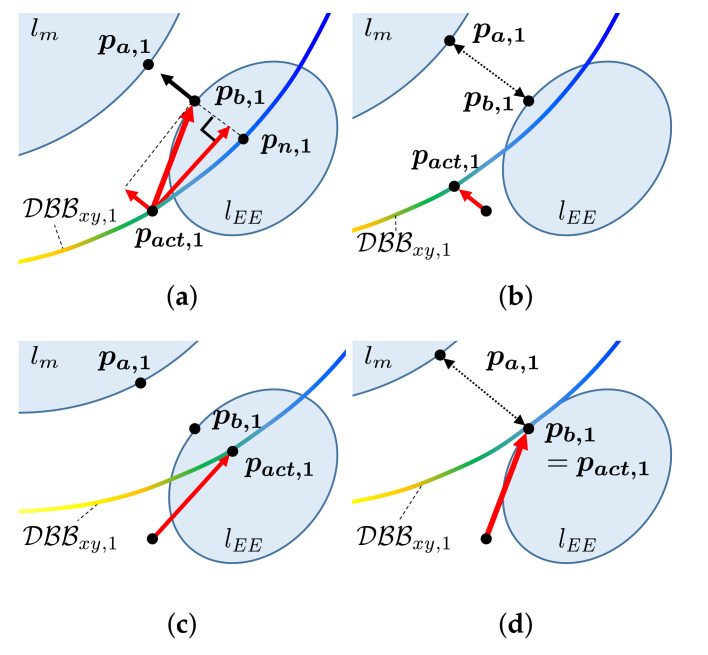
Illustration of the direction of force considering collision between the end-effector $l_{EE}$ and mobile robot $l_{m}$. ${\mathrm{DBB}}_{xy,1}$ denotes the points whose heights are same as that of the closest point $p_{b,1}$ on the end-effector. The points are colored by the score (yellow: high, blue: low). (**a**) The direction of the force starts from the acting point $p_{\mathrm{act},1}$ and points toward $p_{b,1}$. The force can be decomposed into two orthogonal components; (**b**) one of them moves the mobile robot away from the end-effector; (**c**) the other direction places the high-score part of DBB closer to the end-effector; (**d**) by combining these components, self-collision between the end-effector and mobile robot can be avoided.

First, the acting point should be located with the same height of the closest point on the manipulator because the force should act on a horizontal plane to move the mobile robot. Second, the acting point should be selected so that the force has two orthogonal components that play different roles. As shown in Figure 7a, the direction of the force can be decomposed into two orthogonal directions. One is the direction of the line connecting the closest point on the manipulator and the DBB. The other is its normal direction. The former increases the distance between the mobile robot and the manipulator as the DBB moves closer to the manipulator as shown in Figure 7b. On the other hand, as shown in Figure 7c, the latter places the manipulator closer to the DBB with high score in order to avoid selecting the acting point, i.e., the control point of the mobile robot near the singularity and enhance the reachability of the manipulator. Combining two orthogonal components, self-collision between the manipulator and the mobile robot can be avoided in Figure 7d.

**Figure 8:**
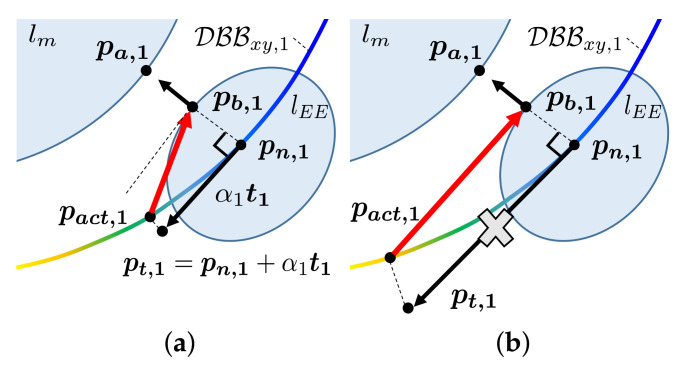
Illustration of finding the acting point. (**a**) The acting point on the DBB is selected as the closest to the point $p_{n,1}$ which is translated along the tangential direction $t_{1}$ with a step size $\alpha_{1}$; (**b**) with a large step size, the generated force may not have a component along the direction connecting the closest point on the manipulator and DBB.

The FindActingPoint function (see Algorithm 3 and Figure 8) finds the acting point that satisfies these requirements. Algorithm 3 operates as follows. First, the TransformToWorld function transforms the points of ${\mathrm{DBB}}_{i}$ to be expressed in the world frame. Next, the FindSameHeight function finds the points in ${\mathrm{DBB}}_{i}$ whose height are same as that of the closest point $p_{b,i}$ on the manipulator, which satisfies the first requirement (see Line 1–3). The obtained points are denoted as ${\mathrm{DBB}}_{xy,i}$. Then, for the second requirement, we calculate the acting point on ${\mathrm{DBB}}_{xy,i}$ that the generated force can have two orthogonal components. Among the points in ${\mathrm{DBB}}_{xy,i}$, the point $p_{n,i}$ closest to the point $p_{b,i}$ is identified (see Line 4). Starting at $p_{n,i}$, the position of point $p_{t,i}$ translated along the tangential direction $t_{i}$ with a step size $\alpha_{i}$ as follows (see Line 5 and Figure 8a):

(12) $$\begin{array}{c} p_{t,i}=p_{n,i}+\alpha_{i}\left(\frac{\nabla S\left(p_{n,i}\right)\cdot t_{i}}{∥\nabla S(p_{n,i})∥}\right)t_{i},\\ t_{i}⊥\left(p_{n,i}-p_{b,i}\right),{∥t_{i}∥}_{2}=1, \end{array}$$

where the inner product of $\nabla S(p_{n,i})$ and $t_{i}$ determines the direction of $t_{i}$ to the higher score of the DBB. The step size $\alpha_{i}$ is calculated as

(13) $$\alpha_{i}\propto \frac{d_{i}}{\left|S(p_{n,i})\right|}.$$

where $d_{i}$ is the distance of the *i*-th link pair of $L_{m}$. Therefore, the acting point $p_{\mathrm{act},i}$ is calculated as that with the shortest distance from $p_{t,i}$ to ${\mathrm{DBB}}_{xy,i}$ (see Line 6). In (13), as the distance between the manipulator and mobile robot decreases, the step size decreases to generate both orthogonal directions of the force. However, a larger step size can be used to proceed more rapidly toward a higher score of the DBB when the distance increases. Additionally, the step size increases as the absolute value of the score of point $p_{n,i}$ decreases to zero, indicating that the point $p_{n,i}$ is near the singularity. To prevent obtaining a step size that is too small or too large as shown in Figure 8b, the step size is bounded by lower and upper limits.
**Algorithm 3** FindActingPoint($q,p_{b,i},{\mathrm{DBB}}_{i}$)1:$z_{b,i}\leftarrow$ height of $p_{b,i}$2:${\mathrm{DBB}}_{i}\leftarrow \mathrm{TransformToWorld}\left(q,{\mathrm{DBB}}_{i}\right)$3:${\mathrm{DBB}}_{xy,i}\leftarrow$ FindSameHeight($z_{b,i}$, ${\mathrm{DBB}}_{i}$)4:$p_{n,i}\leftarrow$ FindMinDistancePoint(${\mathrm{DBB}}_{xy,i}$, $p_{b,i}$)5:$p_{t,i}\leftarrow$ Equation (12) and (13)6:$p_{\mathrm{act},i}\leftarrow$ FindMinDistancePoint(${\mathrm{DBB}}_{xy,i}$, $p_{t,i}$)7:**return**$p_{\mathrm{act},i}$

**Figure 9:**
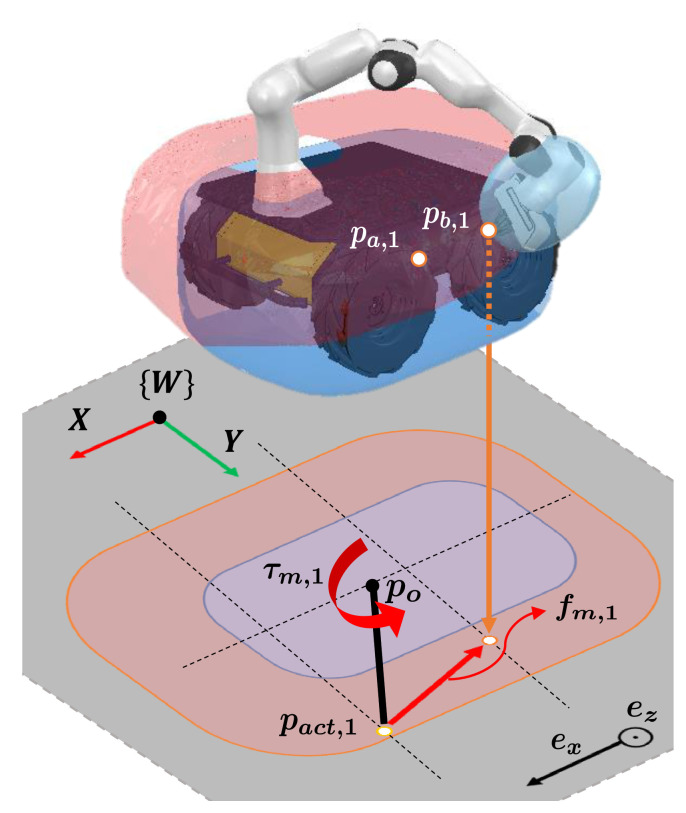
Illustration of generating the desired force and resulting torque for the link pair $L_{m}\left(1\right)$. When the end-effector and mobile robot are close to each other, the force $f_{m,1}$ and resulting torque $\tau_{m,1}$ with respect to the center of the mobile robot, $p_{o}$, are generated. The generated force and resulting torque are projected onto the acceleration directions of the mobile robot under the non-holonomic constraint. The directions are expressed as the unit vectors, $e_{x}$ and $e_{z}$.

After finding the acting point, the GenerateMobAcc function first computes the force as follows:

(14) $$f_{m,i}=f_{max}\left(1-\frac{d_{i}}{d_{b}}\right)\frac{p_{b,i}-p_{\mathrm{act},i}}{∥p_{b,i}-p_{\mathrm{act},i}{∥}_{2}},$$

where $f_{m,i}\in R^{3}$ is the force for the *i*-th link pair and $f_{max}$ is the maximum force. Figure 9 presents the variables in (14) when the link pair $L_{m}\left(1\right)$ is considered.

The resultant force for all link pairs in $L_{m}$ is calculated by adding each force as follows:

(15) $$f_{m}=\sum_{i=1}^{N(L_{m})}f_{m,i},$$

where $f_{m}\in R^{3}$ and $N(L_{m})$ are the resultant force and number of link pairs in $L_{m}$, respectively.

To realize the resultant force, the corresponding accelerations and corresponding Jacobian matrices are derived as follows:

(16) $$\begin{array}{c} {\ddot{\underline{x}}}_{m}={\left[\begin{array}{cc} {\dot{v}}_{d} & {\dot{w}}_{d} \end{array}\right]}^{T}, \end{array}$$

where

(17) $$m{\dot{v}}_{d}=f_{m}\cdot e_{x},$$

(18) $$I{\dot{w}}_{d}=\left(\sum_{i=1}^{N(L_{m})}\left(p_{\mathrm{act},i}-p_{o}\right)\times f_{m,i}\right)\cdot e_{z}.$$

In (17) and (18), m∈R, ${\dot{v}}_{d}\in R$, and $e_{x}\in R^{3}$ are the mass of the mobile robot, desired linear acceleration, and a unit vector perpendicular to the rolling axis of the wheel and pointing forward, respectively. In addition, I∈R, ${\dot{w}}_{d}\in R$, and $e_{z}\in R^{3}$ are the moment of inertia, desired angular acceleration, and a unit vector perpendicular to the ground and pointing upward, respectively. By (17) and (18), the resultant force can be converted into the desired linear and angular accelerations. The Jacobian matrix of the differentially driven mobile robot can be expressed as

(19) $$\begin{array}{c} {\underline{J}}_{m}\;=\;\left[\begin{array}{ccc} J_{m}\; & | & \;O_{2\times n_{m}}\; \end{array}\right],\\ J_{m}\;=\;\left[\begin{array}{cc} \frac{r}{2} & \frac{r}{2}\\ \frac{r}{2b} & -\frac{r}{2b} \end{array}\right], \end{array}$$

where $J_{m}\in R^{2\times 2}$ and $O_{2\times n_{m}}\in R^{2\times n_{m}}$ are the Jacobian matrix of the mobile robot and the zero matrix, respectively.

### 3.2. Generation of the Repulsive Acceleration for the Other Link Pair

To avoid the self-collision of $L_{m}^{c}$, we design a 1-DOF repulsive acceleration to push the link pair away from each other.

Let us consider that the distance of the *j*-th link pair in $L_{m}^{c}$ is less than the buffer distance. The task for avoiding self-collision with the repulsive acceleration ${\ddot{x}}_{r,j}\in R^{1}$ and Jacobian $J_{r,j}\in R^{1\times n}$ is designed as follows.

(20) $${\ddot{x}}_{r,j}={u_{j}}^{T}\left(k_{p}\frac{p_{b,j}-p_{a,j}}{∥p_{b,j}-p_{a,j}{∥}_{2}}-k_{v}\left({\dot{p}}_{b,j}-{\dot{p}}_{a,j}\right)\right),$$

(21) $$\begin{array}{c} J_{r,j}={u_{j}}^{T}\left(J_{b,j}-J_{a,j}\right),\\ u_{j}=\frac{p_{b,j}-p_{a,j}}{∥p_{b,j}-p_{a,j}{∥}_{2}} \end{array}$$

where $k_{p}$ and $k_{v}$ are gains, $u_{j}\in R^{3}$ is the unit vector from $p_{a,j}$ to $p_{b,j}$, and $J_{a,j}$ and $J_{b,j}\in R^{3\times n}$ are translation Jacobian matrices for points $p_{a,j}$ and $p_{b,j}$, respectively. For convenience, we define the link to which $p_{b,j}$ belongs as being farther from the base of the manipulator than the link to which $p_{a,j}$ belongs.

When *k* link pairs in $L_{m}^{c}$ are considered, the repulsive acceleration and Jacobian matrix are stacked as

(22) $${\ddot{\underline{x}}}_{r}={\left[\begin{array}{c} {\ddot{x}}_{r,1},\cdots ,{\ddot{x}}_{r,j},\cdots ,{\ddot{x}}_{r,k}, \end{array}\right]}^{T}$$

(23) $${\underline{J}}_{r}={\left[\begin{array}{c} {J_{r,1}}^{T},\cdots ,{J_{r,j}}^{T},\cdots ,{J_{r,k}}^{T} \end{array}\right]}^{T},$$

where ${\ddot{\underline{x}}}_{r}\in R^{k}$ and ${\underline{J}}_{r}\in R^{k\times n}$ are the stacked accelerations and Jacobians, respectively.

### 3.3. Construction of an Acceleration-Based Task for Self-Collision Avoidance

Based on the obtained accelerations and Jacobians in Section 3.1 and Section 3.2, we construct a task, $T_{sca}$, for avoiding self-collision of all link pairs by stacking them as follows.

(24) $${\ddot{x}}_{\mathrm{sca}}=\left[\begin{array}{c} {\ddot{\underline{x}}}_{m}\\ {\ddot{\underline{x}}}_{r} \end{array}\right],$$

(25) $$J_{\mathrm{sca}}=\left[\begin{array}{c} {\underline{J}}_{m}\\ {\underline{J}}_{r} \end{array}\right],$$

where ${\ddot{x}}_{\mathrm{sca}}\in R^{\left(2+k\right)}$ is the desired acceleration for the avoidance task and $J_{\mathrm{sca}}\in R^{(2+k)\times n}$ is the corresponding Jacobian matrix.

### 3.4. Insertion of the Task in HQP-Based Controller

To insert the designed task, $T_{sca}$, a controller is designed based on the HQP with the continuous task transition approach developed in our previous work [24,25]. HQP is a cascade of QP formulation for dealing with prioritized SoT [32,33]. The main characteristic of the controller with the continuous task transition method is that the continuity of control inputs is always guaranteed even if arbitrary tasks are inserted and removed from the existing SoT. In particular, by using an activation parameter that interpolates feasible solution between existing SoT and new SoT, the method can generate continuous control inputs without modifying the control structure.

We consider the HQP formulation of a single equality task, $T_{2}$, with ${\ddot{x}}_{d_{2}}\in R^{m_{2}}$ and $J_{2}\in R^{m_{2}\times n}$, as follow:

(26) $$\begin{array}{cccc} \;\;\; & \;\;\;\min_{\ddot{q},u,w_{2}}\;\;\; & \;\;\; & ∥w_{2}{∥}_{2},\\ \; & \;s.\;t.\; & \; & M\ddot{q}+C\dot{q}+g=u\\ \; & \; & & J_{2}\ddot{q}+{\dot{J}}_{2}\dot{q}+w_{2}={\ddot{x}}_{d_{2}} \end{array}$$

where $M\in R^{n\times n}$, $C\in R^{n\times n}$, $g\in R^{n}$, and $\dot{q}={\left[\begin{array}{cc} {\dot{q}}_{b}^{T} & {\dot{q}}_{m}^{T} \end{array}\right]}^{T}\in R^{n}$ are the inertia matrix, Coriolis and centrifugal matrix, gravity vector, and joint velocity vector of the non-holonomic mobile manipulator, respectively [34]. In addition, $w_{2}\in R^{m_{2}}$ is a slack variable for $T_{2}$ and $u\in R^{n}$ is the control torque vector for the robot.

**Figure 10:**
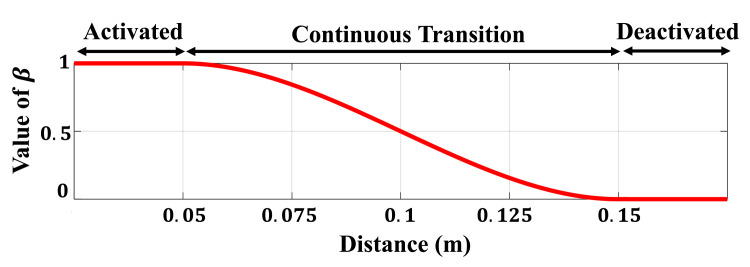
Value of the activation parameter depending on the distance of the link pair.

The activation parameter, β, is determined based on the distance between each link pair. Figure 10 presents the activation trajectory when using a cubic spline to insert $T_{sca}$ smoothly. When the distance is less than the buffer distance of 0.15 m, β gradually increases, and the avoidance task begins to be inserted. In addition, if the distance is less than 0.05 m, β is set to 1 so that the task for avoiding self-collision is fully considered. Because the avoidance tasks for $L_{m}$ and $L_{m}^{c}$ are stacked according to (24), we construct a diagonal matrix B from the activation parameters as follows.

(27) $$B=\left[\begin{array}{ccccc} \beta_{m} & 0 & 0 & \cdots & 0\\ 0 & \beta_{m} & 0 & \cdots & 0\\ 0 & 0 & \beta_{r,1} & \cdots & 0\\ \vdots & \vdots & \vdots & \ddots & 0\\ 0 & 0 & 0 & 0 & \beta_{r,k} \end{array}\right],$$

where $B\in R^{\left(2+k)\times (2+k\right)}$ is the diagonal matrix of the activation parameters, $\beta_{m}$ is the activation parameter for the link pairs of $L_{m}$, and $\beta_{r,j}$ is the activation parameter for the *j*-th link pair in $L_{m}^{c}$. When considering multiple link pairs of $L_{m}$, we choose the maximum value among the activation parameters as

(28) $$\beta_{m}=\max\left(\beta_{1},\cdots ,\beta_{N(L_{m})}\right).$$

Based on the activation parameter matrix B, the HQP formulation for inserting the self-collision avoidance task as the higher-priority task than $T_{2}$ ($T_{sca}≺T_{2}$), can be represented as

(29) $$\begin{array}{cccc} \;\;\; & \;\;\;\min_{\ddot{q},u,w_{2}}\;\;\; & \;\;\; & ∥w_{2}{∥}_{2},\\ \; & \;s.\;t.\; & \; & M\ddot{q}+C\dot{q}+g=u\\ \; & \; & & J_{2}\ddot{q}+{\dot{J}}_{2}\dot{q}+w_{2}={\ddot{x}}_{d_{2}}\\ \; & \; & & J_{\mathrm{sca}}\ddot{q}+{\dot{J}}_{\mathrm{sca}}\dot{q}+\left(I_{2+k}-B\right)J_{\mathrm{sca}}{\ddot{q}}_{2}^{*}+w_{\mathrm{sca}}^{*}=B{\ddot{x}}_{\mathrm{sca}} \end{array}$$

where $w_{\mathrm{sca}}^{*}\in R^{2+k}$ is the optimal slack variable vector for the self-collision avoidance task $T_{sca}$, $I_{2\times k}\in R^{\left(2+k)\times (2+k\right)}$ is an identity matrix, and ${\ddot{q}}_{2}^{*}$ is the optimal solution of (26). Thus, if B is a zero matrix, then the feasible solution of (29) is the same as that of (26). When B is the identity matrix, the solution of (29) is strictly satisfied with the priority order, $T_{sca}≺T_{2}$. In addition, when β gradually increases 0 to 1, the feasible solution of (29) can be derived by interpolating the solution of the HQP of $T_{2}$ and the HQP with $T_{sca}≺T_{2}$. Consequently, the HQP-based controller with the continuous task transition method can insert a self-collision avoidance task without a discontinuous control input.

## 4. Experimental Results

The self-collision avoidance algorithm was verified through various experiments using a differentially driven mobile robot with a 7-DOFs robotic manipulator. The subsections below describe the details of our system configuration and the experimental results for the robot. It is worthwhile to note that the video clips of the experiments described in this paper are available (https://youtu.be/a9dc4Ij71_M), which are applied for not only our robot, but also other differentially driven mobile manipulators to show the generality of the proposed algorithm.

### 4.1. System Overview

Our mobile manipulator consists of the velocity-controlled four-wheel differentially driven mobile robot called Husky (Clearpath Robotics. Co.) and a 7-DOFs robot arm manipulator called Panda (Franka Emika. Co.). The specification of the computer for the controller is an Intel i7 4.2 GHz CPU with 16 GB of RAM and the control frequencies of the manipulator and mobile robot are 1 kHz and 10 Hz, respectively. The desired velocity command for the mobile robot is computed from the desired torque calculated in (29) by applying the admittance control law [35].

### 4.2. Experimental Results

#### 4.2.1. Self-Collision Avoidance While Tracking the Predefined Trajectory

**Figure 11:**
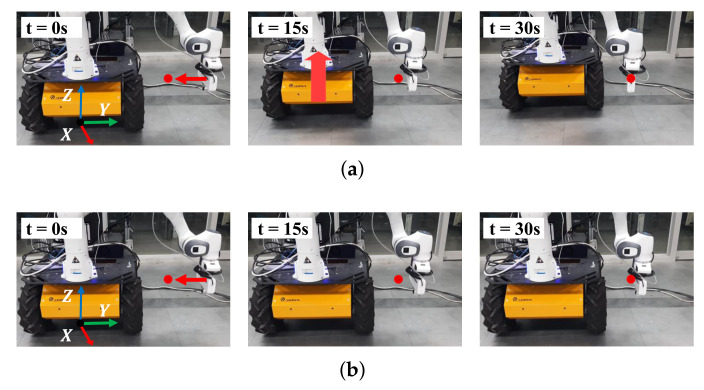
Snapshots during experiments in which the end-effector tracks a predefined trajectory: (**a**) The proposed method generates force to move the mobile robot back, enabling the manipulator to not only avoid self-collision but also reach the target position; (**b**) the repulsive-force based method pushes the manipulator from the mobile robot so that the manipulator cannot reach the target position.

To validate the effectiveness of the proposed method, we conducted a comparative experiment using the repulsive force-based method [19,20]. This experiment was designed for the end-effector to track a predefined trajectory that approaches the mobile robot. The task for trajectory tracking of the end-effector is denoted as $T_{ee}\in R^{6}$ and the task for the repulsive force-based method is denoted as $T_{rep}\in R^{3}$. The target position is −0.2 m along the Y-axis from the end-effector. The left snapshots in Figure 11a,b shows the initial positions. The red dots and arrows depict the target position and desired trajectory, respectively. The trajectory was generated for a time period of 30 s using a cubic spline function.

**Figure 12:**
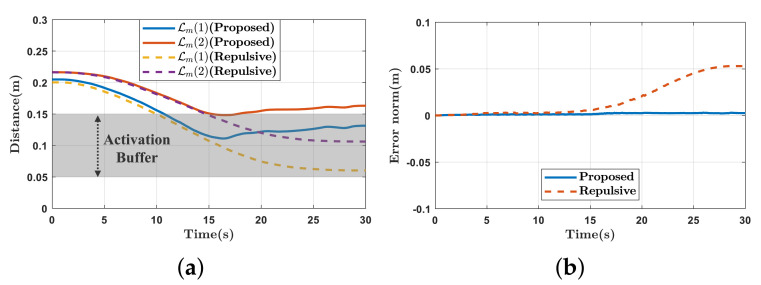
Experimental results of self-collision avoidance while tracking the predefined trajectory. (**a**) Distances of the link pairs ($L_{m}\left(1\right),L_{m}\left(2\right)$); (**b**) the norm of the position error.

The experimental results are presented in Figure 11 and Figure 12. In Figure 11a, as the end-effector moves close to the mobile robot, force is exerted to move the mobile robot back. As a result of the force, the end-effector reaches the target position while avoiding self-collision. In contrast, in Figure 11b, because repulsive force is generated to push the end-effector away from the mobile robot, self-collision is avoided, but the end-effector can not reach the target position. The distances between the link pairs are shown in Figure 12a. Because the distances are less than 0.15 m, the self-collision avoidance tasks ($T_{sca}$ and $T_{rep}$) are inserted continuously with top priority. In Figure 12b, the repulsive force-based method has a position error, while the proposed method does not.

#### 4.2.2. Self-Collision Avoidance While Manually Guiding the End-Effector

**Figure 13:**
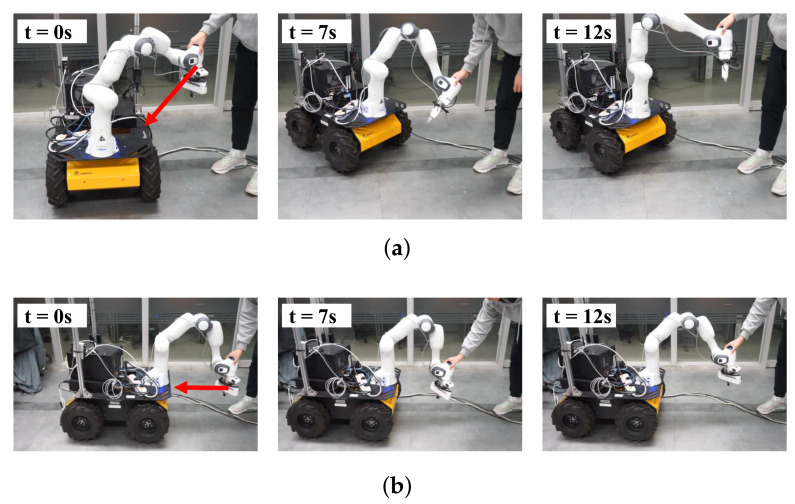
(**a**) The snapshots during the experiment that the manipulator approaches the mobile from the lateral direction; (**b**) the snapshots during the experiment that the manipulator approaches the mobile robot from the front direction. Red arrows show the guiding directions.

In this experiment, the end-effector was manually guided by an operator to approach the mobile robot to validate reactive self-collision avoidance during human–robot interaction. In the initial state, no tasks are executed other than the gravity compensation of the manipulator. Two directions are considered: the lateral direction and the front direction. The left snapshots in Figure 13 show the initial positions of the mobile manipulator and the guiding directions are depicted by red arrows.

**Figure 14:**
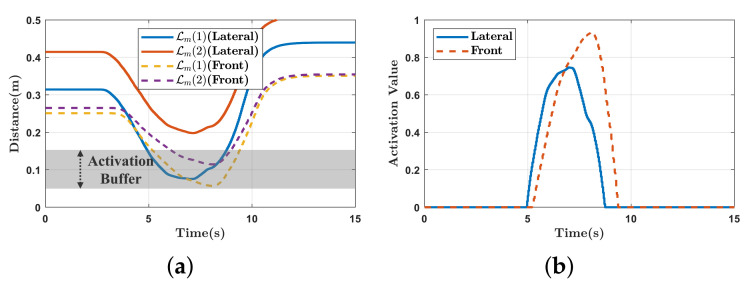
Experimental results of self-collision avoidance while manually guiding the end-effector. (**a**) The distances between the link pairs ($L_{m}\left(1\right),L_{m}\left(2\right)$); (**b**) the value of the activation parameter.

**Figure 15:**
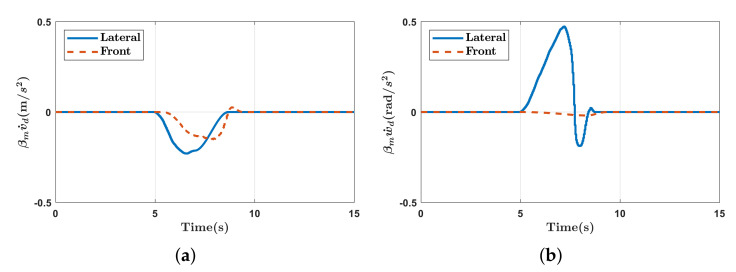
Experimental results of self-collision avoidance while manually guiding the end-effector. (**a**) The desired linear accelerations multiplied by activation parameter; (**b**) the desired angular accelerations multiplied by activation parameter.

As shown in Figure 13a, self-collision between the manipulator and mobile robot are avoided by generating a force exerted on the mobile robot. As shown in Figure 13b, as the manipulator approaches the mobile robot, the mobile robot moves back to avoid self-collision. Figure 14 presents the distances between the links of the manipulator and mobile robot and the values of the activation parameter. As the distance decreases below the buffer distance of 0.15 m, the value of the activation parameter increases accordingly and the self-collision avoidance task, $T_{sca}$, is inserted continuously as shown in Figure 14b. In Figure 15, the command values of the linear and angular accelerations of the HQP-based controller are plotted. Therefore, self-collision can be avoided regardless of the approach direction of the manipulator, which is an advantage over existing methods [11,20] that do not consider the non-holonomic constraint of the differentially driven mobile robot.

#### 4.2.3. Extension to Obstacle Avoidance When Opening the Refrigerator

**Figure 16:**
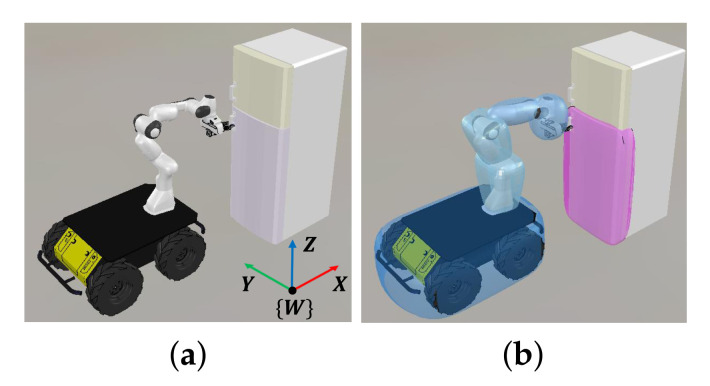
(**a**) Illustration of the scenario of the mobile manipulator opening a refrigerator; (**b**) collision models including the door of the refrigerator are shown. The collision model for the door is colored with magenta.

In this subsection, we extend our method to obstacle avoidance. The proposed method was tested in a reactive scenario representing a typical example of mobile manipulation. We consider the scenario of opening the refrigerator as shown in Figure 16a. We assume that the end-effector achieves a fixed grasp on the door of the refrigerator, meaning there is no relative motion between them. In this respect, collision between the door and mobile robot is considered. We used a hyper-ellipsoid to design a collision model for the door as shown in Figure 16b.

To open the refrigerator, a control strategy based on adaptive control [36,37] was utilized. The strategy estimates the radial direction of the door based on the force measured at the end-effector so that the end-effector can open the door even with the incomplete knowledge regarding door models. The strategy uses only the manipulator to open the door, meaning the robot may collide with the door depending on the initial pose of the mobile robot. We validated our extension to obstacle avoidance by comparing the results of experiment with and without obstacle avoidance. The scenario of opening the refrigerator was validated in both simulation and experiment.

**Figure 17:**
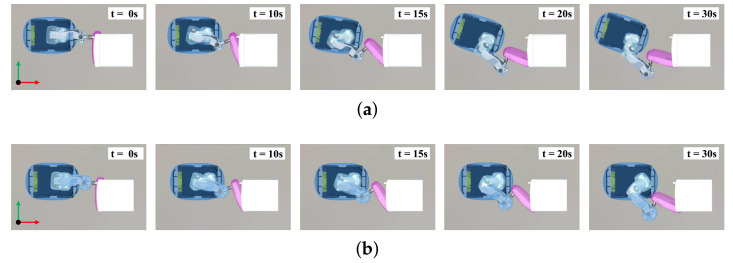
Simulation results of opening a refrigerator. (**a**) Snapshots of opening a refrigerator with obstacle avoidance; (**b**) snapshots of opening a refrigerator without obstacle avoidance.

**Figure 18:**
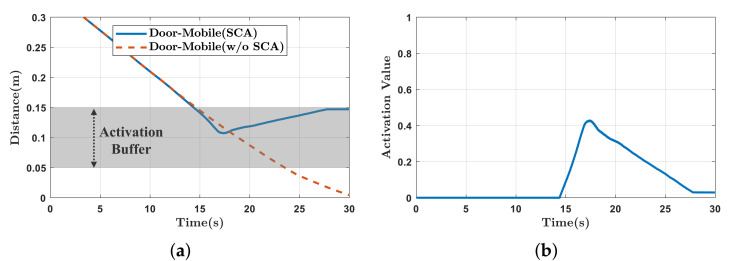
Simulation results of opening the refrigerator. (**a**) The distance between the door and the mobile robot; (**b**) the value of the activation parameter.

The simulation results are presented in Figure 17 and Figure 18. In Figure 17a, the mobile robot moves back and turns clockwise as the door moves closer to the mobile robot. In contrast, the door collides with the mobile robot at 30 s in Figure 17b. As the distance between the door and mobile robot is less than the buffer distance in Figure 18a, the obstacle avoidance task is inserted continuously as shown in Figure 18b.

**Figure 19:**
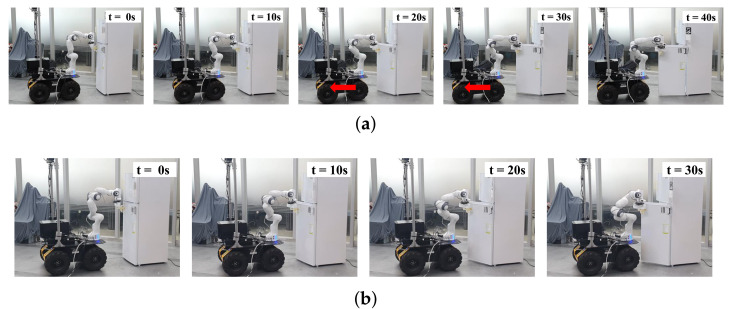
Experimental results of opening a refrigerator. (**a**) Snapshots of opening a refrigerator with obstacle avoidance; (**b**) snapshots of opening a refrigerator without obstacle avoidance.

The experimental results are presented in Figure 19. As shown in Figure 19a, as the distance between the door and robot decreases, the mobile robot begins to move back at 20 s and the manipulator opens the door completely at 40 s, while avoiding collision. In contrast, in Figure 19b, the manipulator stops opening the door at 30 s because the robot is about to collide with the door.

### 4.3. Discussion

The experimental results in Section 4.2 demonstrate that the proposed method can place the manipulator outside the DBB. Specifically, the proposed method has the following advantages. First, the force can always generate motion for the differentially driven mobile robot with non-holonomic constraint as shown in Section 4.2.2. This is because the acting point is selected such that it is away from the singularity of the mobile robot. Second, the proposed method can be applied to holonomic mobile manipulators if the score of the DBB is designed to include only the reachability of the manipulator. Finally, command values are free from chattering and vibration problems caused by the mobile robot unlike repulsive force-based method [38]. This is because the continuous task transition of (29) can calculate continuous control input. Therefore, the desired accelerations of the mobile robot are smooth, as shown in Figure 15.

From a practical perspective, a trade-off relationship exists between the density of the DBB and the discontinuous position of the acting point. The denser the DBB, the more computational cost increases. However, with a denser DBB, the position of the acting point can be obtained more continuously. According to our practical experience, the proper number of points in the DBB is approximately 50,000 for running the algorithm at a control frequency of 1 kHz.

## 5. Conclusions

We presented a reactive self-collision avoidance algorithm for differentially driven mobile manipulator. The proposed algorithm generates a force exerted on a mobile robot in a direction that the manipulator is placed outside the DBB. The force is designed based on the concept of the DBB and its score measurement. The score consists of two components, the determinant value of the Jacobian matrix of differentially driven mobile robot and the reachability of the manipulator. After calculating the score for each point on the DBB, a point with high score is selected as the acting point of force, which means that the point is away from the singularity of differentially driven mobile robot and has high reachability of the manipulator. Based on the force and resulting torque, an avoidance task is formulated and inserted into the HQP-based controller with a continuous task transition algorithm. The results of several experiments validated the proposed self-collision avoidance algorithm. Our future work will involve extending the proposed method to other mobile platforms like car-like robots and developing collision avoidance method by using external sensors like cameras. Additionally, we will apply the proposed algorithm to a wider range of mobile manipulation tasks that needs to detect collision in dynamic and unstructured environment.

## Figures and Tables

**Table 1:** Notation and symbols.

Symbol	Description
$<l_{a},l_{b}>$	Pair of the links *a* and *b*
$L=L_{m}\cup L_{m}^{c}$	Set of potentially colliding link pairs
$L_{m}$	Subset of L including the mobile robot
$L_{m}^{c}$	Complement set of $L_{m}$
${\mathrm{DBB}}_{i}$	DBB of *i*-th link pair in $L_{m}$
*n*	DOFs of the mobile manipulator
$n_{m}$	DOFs of the manipulator
$T_{j}$	*j*-th equality or inequality task
$T_{j}≺T_{j+1}$	$T_{j}$ has higher priority than $T_{j+1}$

## Data Availability

Not applicable.

## Abbreviations

DBB	Distance Buffer Border
DOFs	Degrees Of Freedom
HQP	Hierarchical Quadratic Programming
SoT	Stack of Tasks
